# Immune Response in Moderate to Critical Breakthrough COVID-19 Infection After mRNA Vaccination

**DOI:** 10.3389/fimmu.2022.816220

**Published:** 2022-01-25

**Authors:** Krystallenia Paniskaki, Moritz Anft, Toni L. Meister, Corinna Marheinecke, Stephanie Pfaender, Sarah Skrzypczyk, Felix S. Seibert, Constantin J. Thieme, Margarethe J. Konik, Sebastian Dolff, Olympia Anastasiou, Bodo Holzer, Ulf Dittmer, Christine Queren, Lutz Fricke, Hana Rohn, Timm H. Westhoff, Oliver Witzke, Ulrik Stervbo, Toralf Roch, Nina Babel

**Affiliations:** ^1^ Department of Infectious Diseases, West German Centre of Infectious Diseases, University Hospital Essen, University Duisburg-Essen, Essen, Germany; ^2^ Center for Translational Medicine and Immune Diagnostics Laboratory, Medical Department I, Marien Hospital Herne, University Hospital of the Ruhr-University Bochum, Herne, Germany; ^3^ Department of Molecular and Medical Virology, Ruhr-University Bochum, Bochum, Germany; ^4^ Medical Department I, Marien Hospital Herne, University Hospital of the Ruhr-University Bochum, Herne, Germany; ^5^ Berlin Institute of Health at Charité – University Clinic Berlin, BIH Center for Regenerative Therapies (BCRT), Berlin, Germany; ^6^ Institute for Virology, University Hospital Essen, University of Duisburg-Essen, Essen, Germany; ^7^ Dialysis Center Dialyse Bochum, Bochum, Germany

**Keywords:** SARS-CoV-2, vaccine, mRNA, breakthrough infection, T cells, neutralizing antibodies

## Abstract

SARS-CoV-2 variants of concern (VOCs) can trigger severe endemic waves and vaccine breakthrough infections (VBI). We analyzed the cellular and humoral immune response in 8 patients infected with the alpha variant, resulting in moderate to fatal COVID-19 disease manifestation, after double mRNA-based anti-SARS-CoV-2 vaccination. In contrast to the uninfected vaccinated control cohort, the diseased individuals had no detectable high-avidity spike (S)-reactive CD4+ and CD8+ T cells against the alpha variant and wild type (WT) at disease onset, whereas a robust CD4+ T-cell response against the N- and M-proteins was generated. Furthermore, a delayed alpha S-reactive high-avidity CD4+ T-cell response was mounted during disease progression. Compared to the vaccinated control donors, these patients also had lower neutralizing antibody titers against the alpha variant at disease onset. The delayed development of alpha S-specific cellular and humoral immunity upon VBI indicates reduced immunogenicity against the S-protein of the alpha VOC, while there was a higher and earlier N- and M-reactive T-cell response. Our findings do not undermine the current vaccination strategies but underline a potential need for the inclusion of VBI patients in alternative vaccination strategies and additional antigenic targets in next-generation SARS-CoV-2 vaccines.

## Introduction

A year since the first anti-SARS-CoV-2 vaccine was approved for use (1, 2), increasing concerns have arisen regarding the effectiveness of the existing SARS-CoV-2 vaccines against variants of concern (VOCs). Current research findings are contradictory regarding the protective potential of adaptive immunity induced either after SARS-CoV-2 vaccination or natural infection against VOCs. Independent groups have shown that CD4+ and CD8+ T-cell responses in convalescent COVID-19 subjects and SARS-CoV-2 mRNA vaccinees are not substantially affected by mutations found in the SARS-CoV-2 variants (3–5) and that the impaired neutralization capacity of sera against the alpha VOC after two BNT162b2 doses seems to be negligible (6–9).

However, other reports suggest a significant reduction in SARS-CoV-2 neutralization by vaccine sera against VOCs (10–14). For example, a significant reduction in neutralization titers by vaccine sera was observed when mutation E484K was present alongside the alpha variant S-protein, especially in combination with N501Y and K417N8 (7, 10, 15). Similarly, vaccine and convalescent sera showed reduced neutralization of kappa and delta variants (16, 17).

Vaccine breakthrough infections (VBI), mainly with VOCs, has been observed in different cohorts, including healthy young individuals and elderly patients with significant comorbidities (18–20). Taking into consideration that the anti-SARS-CoV-2 vaccines were shown to protect efficiently against critical and fatal COVID-19 manifestations, it is rather unusual to observe critical COVID-19 cases or patients who died due to COVID-19 in fully vaccinated population/cohorts (21). In this study, we analyzed the specific SARS-CoV-2 T-cell and humoral immune responses of 8 patients who developed COVID-19 with moderate to fatal disease severity despite being fully vaccinated against SARS-CoV-2 with one of the mRNA vaccines. The vaccinated control group comprised 5 healthy donors, who were double immunized against the SARS-CoV-2 virus.

## Materials and Methods

### Study Participants

We used PBMCs and serological samples from 8 patients with COVID-19 disease who were previously inoculated with two shots of an approved SARS-CoV-2 vaccine (further referred as VBI). Three control groups were recruited: a) 5 healthy individuals who were vaccinated against SARS-CoV-2 virus (vac-healthy), b) 14 unvaccinated COVID-19 diseased adults (no-vac COVID) and c) 8 unvaccinated and unexposed to SARS-CoV-2 individuals (no-vac unexposed). The study was approved by the Ethics Committee of the Ruhr University Bochum (20–7126) and University Hospital Essen (20-9753-BO). Written informed consent was obtained from all participants. Demographic and clinical characteristics are provided in **Tables S1**–**3**.

Where available, the blood samples of the VBI and no-vac COVID diseased study groups were collected during follow-up visits: T1, third day since SARS-CoV-2 diagnosis; T2, 7 days since the diagnosis; and T3, 14 days since T1. The vac-healthy control group consisted of 5 healthy donors immunized twice with the BNT162b2 vaccine. Blood samples from the vac-healthy cohort were collected on the day of the first vaccination before the vaccine was administered (Ta), and 14 days after the second vaccination (Tb). The recruitment and collection of PBMCs from the unvaccinated and unexposed to SARS-CoV-2 individuals took place between 2015 and 2017.

### Preparation of PBMCs

As previously described, peripheral blood was collected in S-Monovette K3 EDTA blood collection tubes (Sarstedt) (22). Collected blood was prediluted in PBS/BSA (Gibco) at a 1:1 ratio and underlaid with 15 mL of Ficoll-Paque Plus (GE Healthcare). Tubes were centrifuged at 800g for 20 min at room temperature. Isolated PBMCs were washed twice with PBS/BSA and stored at -80°C until use. The cryopreserved PBMCs were thawed by incubating cryovials 2-3 minutes at 37°C in bead bath, washed twice in 37°C RPMI 1640 media (Life Technologies) supplemented with 1% penicillin-streptomycin-glutamine (Sigma-Aldrich), and 10% fetal calf serum (FCS) (PAN-Biotech) medium, and incubated overnight at 37°C.

### Flow Cytometry- Measurement of SARS-CoV-2 Reactive T Cells

As previously described, PBMCs were plated in 96-U-Well plates in RPMI 1640 media (Life Technologies) (22, 23). Each well was stimulated with one of the following SARS-CoV-2 proteins spanning *in silico* predicted immunodominant parts of the; WT Spike SARS-CoV-2, B.1.1.7 D614G Spike mutant (JPT Peptide Technologies), the complete sequence of the N- or M-protein (Miltenyi Biotec) or left untreated as a control for 16 h. The proteins were dissolved per manufacturer’s directions in sterile water or DMSO regarding the Miltenyi Biotec peptides and JPT Peptide Technologies respectively. As a positive control, cells were stimulated with staphylococcal enterotoxin B (1 μg/mL, Sigma-Aldrich). After 2 h, brefeldin A (1 μg/mL, Sigma-Aldrich) was added. Detailed listing of the antibody panels for general phenotyping and T cell activation *ex vivo* is in **Table S4**. The PBMCs stimulated overnight were stained with optimal concentrations of antibodies for 10 min at room temperature in the dark. Stained cells were washed twice with PBS/BSA before preparation for intracellular staining using the Intracellular Fixation & Permeabilization Buffer Set (Thermo Fisher Scientific) as per the manufacturer’s instructions. Fixed and permeabilized cells were stained for 30 min at room temperature in the dark with an optimal dilution of antibodies against the intracellular antigen. All samples were immediately acquired on a CytoFLEX flow cytometer (Beckman Coulter). Quality control was performed daily using the recommended CytoFLEX daily QC fluorospheres (Beckman Coulter). No modification to the compensation matrices was required throughout the study. Antigen-reactive responses were considered positive after the non-reactive background was subtracted, and more than 0.01% were detectable. Negative values were set to zero.

### SARS-CoV-2 IgG Antibody Titers

Peripheral blood was collected in S-Monovette Z-Gel (Sarstedt). SARS-CoV-2 IgG titers were analyzed in purified serum using a SARS-CoV-2 IgG kit (Euroimmun, Lübeck, Germany). The test was performed according to the manufacturer’s instructions. Briefly, serum samples were diluted 1:100 and added to plates coated with recombinant SARS-CoV-2 antigen. Bound SARS-Cov-2 S1 protein-reactive IgG was detected by a horseradish peroxidase (HRP)-conjugated anti-human IgG. The absorbance was read on a microplate reader at 450 nm with reference at 620 nm, and evaluated as the ratio of the absorbance of the sample to the absorbance of the internal standard.

### SARS-CoV-2 WT and Alpha Neutralization Assay

As previously described (24), for the virus neutralization assay, sera were incubated for 30 min at 56°C in order to inactivate complement factors. Single cycle VSV∗ΔG(FLuc) pseudoviruses bearing the SARS-CoV-2 Spike (D614G) protein or SARS-CoV-2 B.1.1.7. (Alpha) Spike protein, or SARS-CoV-2 WT virus or SARS-CoV-2 B.1.1.7. (Alpha) virus in the envelope were incubated with quadruplicates of two-fold serial dilutions of immune sera in 96-well plates prior to infection of Vero E6 cells (1x10^4^ cells/well) in DMEM + 10% FBS (Life Technologies). At 18 hours post infection, firefly luciferase (FLuc) reporter activity was determined as previously described S5 using a CentroXS LB960 (Berthold) and the reciprocal antibody dilution causing 50% inhibition of the luciferase reporter was calculated (PVND50).

### Statistics

Flow cytometry data were analyzed using FlowJo version 10.6.2 (BD Biosciences); gating strategy is presented in **Figure S1**. For the analysis of anti-SARS-CoV-2 T cells, a threshold of 0.01% was employed to define a detectable response. Single stains and fluorescence-minus-one controls were used for gating. Gates of each study participant were adjusted according to the negative control. CD4+ T cells expressing CD154 and CD137 and CD8+ T cells expressing CD137 were defined as reactive T cells. Statistical analysis was performed using GraphPad Prism v7. Categorical variables are summarized as numbers and frequencies; quantitative variables are reported as median and interquartile range. Normality Tests were performed with D’Agostino & Pearson test. All applied statistical tests are two-sided. Frequencies of SARS-CoV-2-protein reactive T cells in the diseased study group and the control groups were compared using exact two-tailed Mann-Whitney Test, and for grouped data the Mann-Whitney Test. For paired data the Wilcoxon matched pairs test was used. The age between the two cohorts was compared using unpaired two-tailed t-test, and gender was compared using two-tailed Fisher’s exact test. p values below 0.05 were considered significant; only significant p values are reported in the figures. p values were not corrected for multiple testing, as this study was of an exploratory nature.

### Study Approval

The study was approved by the Ethics Committee of the Ruhr University Bochum (20–7126) and University Hospital Essen (20-9753-BO). Written informed consent was obtained from all participants.

## Results

### Characterization of the VBI Study Group

Our study group comprised 8 individuals who developed moderate to fatal COVID-19 disease after receiving a double anti-SARS-CoV-2 vaccination, hereafter referred to as the VBI diseased study group. The COVID-19 disease severity classification system of the WHO was applied to define the disease severity of the diseased study patients. Two (24%) patients presented moderate disease severity, three (38%) presented severe pneumonia, and three (38%) were critically ill and admitted to the intensive care unit. Two out of 8 patients succumbed to COVID-19 disease. The diagnosis of SARS-CoV-2 infection occurred a median of 31.5 days after the second vaccination (range 10-75 days). Six patients (74%) received the BNT162b2 vaccine, and two patients (26%) were inoculated with mRNA-1273. The three patients with critical disease manifestations were vaccinated with the BNT162b2 vaccine. SARS-CoV-2 RNA sequencing of nasal swabs detected the S-N501Y mutation with H69/V70 deletion in 100% of the VBI diseased study group, hereafter referred to as the Alpha variant, according to WHO nomenclature (https://www.who.int/en/activities/tracking-SARS-CoV-2-variants/). All patients were negative for the S-E484K mutation. To assess the viral load of the VBI diseased study group at the time of SARS-CoV-2 diagnosis, we determined the Ct of RT–PCR of the nasal swabs. We documented a median Ct of 19.96 (range 15-25). The median age of the VBI diseased study group was 80.5 years (range 28-82 years), whereas the vaccinated control cohort was significantly younger, with a median age of 31 years (range 27-34 years, p=0.003 two tailed unpaired t test). The VBI diseased study group comprised of 38% (n=3) and 62% (n=5) male and female patients, respectively. The demographic and clinical characteristics of the study cohorts are presented in **Tables S1**–**S3**.

### Robust SARS-CoV-2 Reactive CD4+ T-Cell Response Against WT and Alpha S Protein Among the Vaccinated Control Group

We initially assessed the evolution of SARS-CoV-2 reactive CD4+ T-cell responses in the vac-healthy study group before the 1^st^ (Ta) and after the 2^nd^ (Tb) vaccination. CD4+ T cells were quantified by flow cytometry using CD154 and CD137 co-expression as markers for antigen-reactive T cells (gating strategy **Figure S1**). SARS-CoV-2 CD4+ T cells reactive to the S wild-type protein are referred to hereafter as WT S-reactive CD4+ T cells, whereas those reactive against the S alpha protein are referred to as alpha S-reactive CD4+ T cells. As expected, a significant increase in the S-protein-reactive CD4+ T-cell response against the WT S-protein and the alpha variant from Ta to Tb was detected (**Figure S2A**).

T cells interact with the cognate antigen presented on the MHC through the T cell receptor. Higher T cell receptor avidity results in a stronger activation signal, which in turn leads to a proportionate reduction in the T cell receptor complex (25, 26). It is therefore possible to elucidate the T cell receptor avidity toward the activating protein by evaluating the CD3 expression levels (27, 28). CD3 expression levels can be assessed by surface (29) or intracellular staining (30, 31). Therefore, we analyzed S-reactive CD4+CD154+CD137+ T cells for intracellular CD3 expression. Similar to the general response, a statistically significant increase in the CD3low response among CD4+CD154+CD137+ T cells on Tb against the two tested proteins was observed (**Figure S2B**).

### VBI Patients Failed to Develop High-Avidity S-Reactive CD4+ T Cells Against the Alpha S and WT S Proteins at Disease Onset

**Figure 1:**
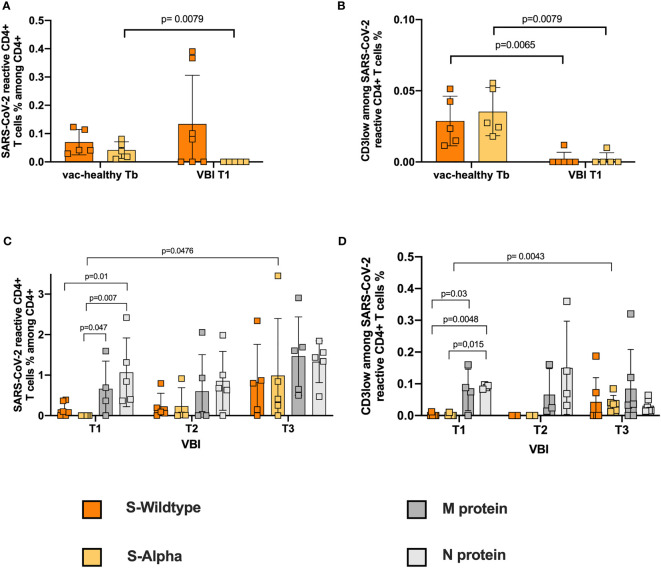
Failure to develop S-reactive CD4+ T cells against the Alpha variant and WT S-reactive CD4+ T cells with high avidity at disease onset. Characterization of SARS-CoV-2 S-reactive CD4+ T cells in diseased and healthy subjects. Blood samples of 8 diseased patients with SARS-CoV-2 breakthrough infection and 5 healthy vaccinated patients were stimulated with SARS-CoV-2 S-WT, S-Alpha, M- and N-proteins, and analyzed by flow cytometry. **(A, B)** S-WT and S-Alpha reactive CD4+ T cells and their avidity between control at Tb and diseased at T1. Absence of Alpha reactive CD4+ T cells at disease onset, accompanied by presence of WT reactive CD4+ T cells with no avidity. **(C, D)** Quantification of S-WT, S-Alpha, M- and N-reactive CD4+ T cells among the diseased. Statistically significant strong frequencies of M- and N- reactive T cells compared to S-WT and S-Alpha reactive T cells at disease onset, with significant higher avidity. Generation of reactive CD4+ T cell response against all 4 SARS-CoV-2 proteins at T3. Reactive SARS-CoV-2 CD4+ T cells are defined as CD4+CD154+CD137+ cells. The avidity was determined by detecting CD3low+CD4+ T cells among CD4+CD154+CD137+. Negative controls were subtracted from reactive stimulated samples to exclude unreactive activation. Bars show mean and standard deviation. Paired data was compared with Wilcoxon matched pairs test, whereas unpaired data with Mann-Whitney-test. P<0.05 was considered significant, only significant p values are documented in the figures.

Next, we analyzed the S-reactive T-cell response in VBI patients and the vac-healthy cohort. While the frequencies of WT S-protein-reactive CD4+ T cells did not show any significant difference between the two groups, the alpha S-specific CD4+ T-cell response showed significantly higher frequencies in the vac-healthy group than in the VBI group at disease onset (**Figure 1A**). Assessing avidity showed that the VBI patients demonstrated significantly lower frequencies of WT and alpha S-reactive CD4+ T cells than the vac-healthy group (**Figure 1B**).

### Robust SARS-CoV-2 CD4+ T-Cell Response Against N and M Proteins at Disease Onset

Previously, we and others showed that comprehensive assessment of SARS-CoV-2 T cell immunity against not only S protein but also M and N proteins is required to avoid underestimation of the T-cell response after SARS-CoV-2 infection (32–34). To this end, we evaluated T cells reactive against M and N SARS-CoV-2 in the VBI cohort. At disease onset (T1), the N- and M-reactive CD4+ T-cell response of the VBI cohort was robust and significantly stronger than the alpha S-reactive CD4+ T-cell response (**Figure 1C**). Furthermore, N-reactive CD4+ T cells presented significantly higher frequencies than WT S-reactive CD4+ T cells on T1 (**Figure 1C**). In the clinical follow-up (T3), a significant increase in the alpha S-reactive CD4+ T-cell response was observed compared to the initial time point (T1). Moreover, at T3, a robust SARS-CoV-2 reactive CD4+ T-cell response against all four SARS-CoV-2 proteins was observed, without significant statistical differentiations among them (**Figure 1C**).

Furthermore, we analyzed the frequencies of high-avidity SARS-CoV-2 CD4+ T cells reactive against all four proteins. At T1, N-reactive CD4+ T cells showed a significantly higher frequency than CD4+ T cells reactive against WT and alpha S-protein. In addition, the frequencies of high-avidity M-reactive CD4+ T cells were significantly higher than those of alpha S-reactive CD4+ T cells (**Figure 1D**). Analysis of high-avidity S-reactive T cells in follow-up demonstrated a significant increase in alpha S-reactive CD4+ T cells at T3 compared to T1. The frequencies of SARS-COV-2 CD4+ T cells reactive against all four proteins were not different at T3 (**Figure 1D**).

The production of IL2, TNFα, and IFNγ by SARS-CoV-2-reactive CD4+ T cells presented a similar pattern. The VBI cohort failed to mount cytokine-producing T cells reactive against the WT S-protein and alpha VOCs at disease onset (**Figure S3**). However, IL2- and TNFα-producing N-reactive CD4+ T cells were detectable at significantly higher frequencies than alpha S-reactive CD4+ T cells, followed by M-reactive CD4+ T cells, which had high frequencies but did not reach statistical significance (**Figures S3A, B**). In the course of COVID-19 disease (T3), IL2, TNFα, and IFNγ production against all four proteins increased (**Figure S3**).

Taking into consideration that in three out of eight VBI patients (P2, P4, P6) the SARS-CoV-2 infection was diagnosed 10 days after the administration of 2^nd^ vaccination, a SARS-CoV-2 contact/infection prior to the 2nd vaccination could not be excluded. Therefore, we re-analyzed the frequencies of SARS-CoV-2 reactive CD4+ T cells and the CD3low population among them excluding the patients P2, P4 and P6. As shown in **Figure S4**, the absence of WT and alpha S-reactive CD4+ T cells at T1, accompanied by high frequencies of N- and M-reactive CD4+ T cells with functional avidity at T1 could be re-confirmed and supported the associations found and presented in **Figure 1**.

### Failure to Induce a Strong SARS-CoV-2 Reactive Cytotoxic CD8+ Response

**Figure 2:**
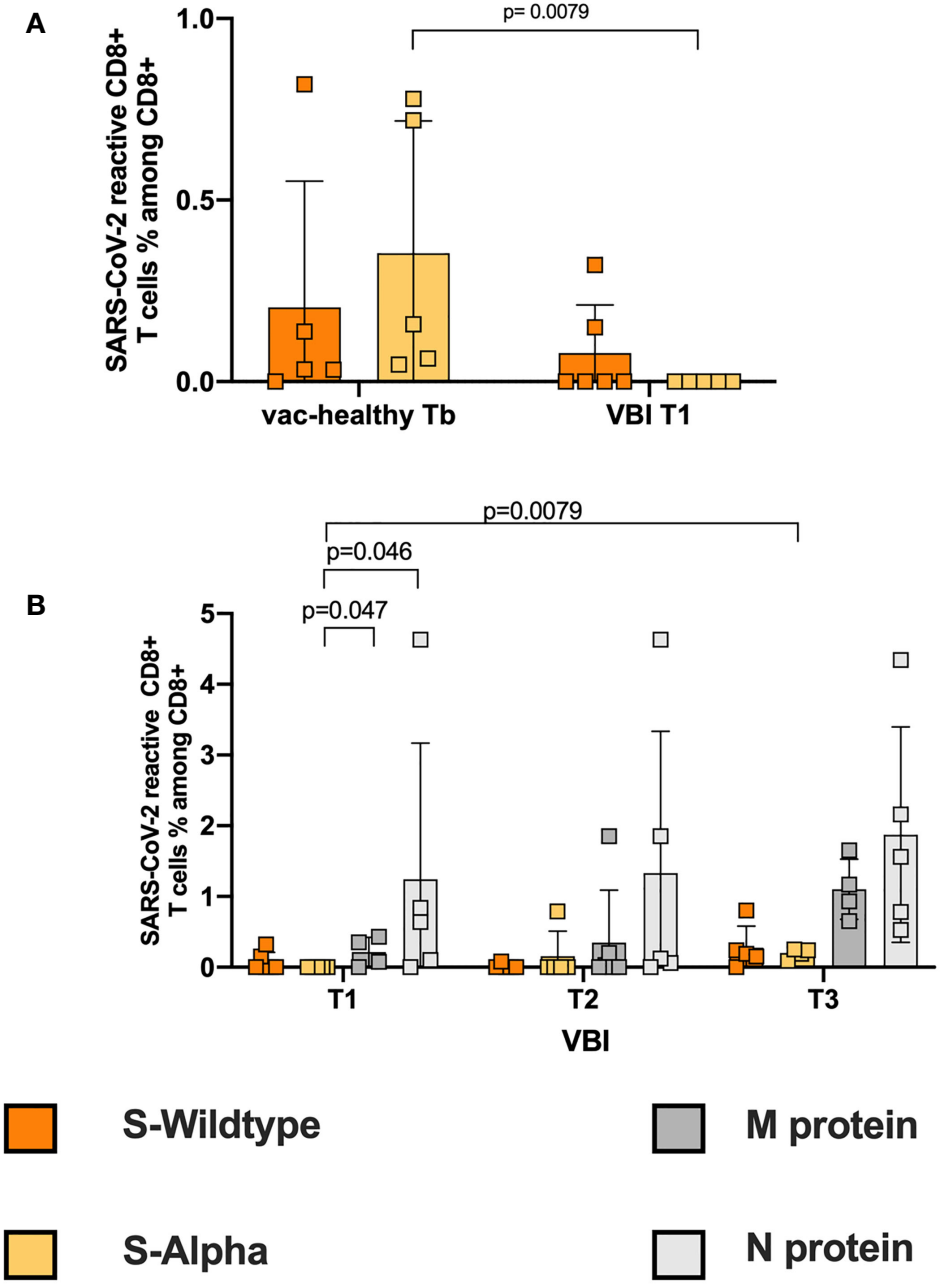
Failure to induce a strong SARS-CoV-2 reactive cytotoxic CD8+ response. **(A)** Correlation of S-WT and S-Alpha reactive CD8+ T cells between controls at Tb and VBI patients at T1. In the VBI cohort there is an absence of S-Alpha reactive CD8+ T cells at disease onset, accompanied by decreased frequencies of S-WT reactive CD4+ T cells, compared to the control. **(B)** Correlation of S-WT, S-Alpha, M- and N-reactive CD8+ T cells among VBI. There are statistically significant higher frequencies of M- and N- reactive T cells compared to S-Alpha reactive T cells on disease onset.

Cytotoxic CD8+ T cells are one of the main components of antiviral responses. First, we evaluated the SARS-CoV-2 reactive CD8+ T-cell response of the vac-healthy group. We defined CD8+CD137+ T cells as reactive SARS-CoV-2 T cells (gating strategy, **Figure S1**). The vac-healthy cohort demonstrated stronger SARS-CoV-2 reactive CD8+ T cell frequencies against the S-WT and the alpha variant after the 2^nd^ vaccination (Tb) compared to Ta. This difference however did not reach statistical significance (Fig S2B). However, at disease onset, the VBI cohort failed to mount an alpha S-reactive CD8+ T-cell response and showed a significantly lower frequency compared to the vac-healthy (**Figure 2A**). The frequencies of both M- and N-reactive CD8+ T cells were significantly higher than those of alpha-S (**Figure 2B**). During the course of the disease (T3), the VBI diseased group managed to mount a significantly stronger alpha S-reactive CD8+ T-cell response compared to T1 (**Figure 2B**).

### Impaired WT and Alpha Neutralizing Antibody Production at Disease Onset

SARS-CoV-2-reactive antibodies produced by B cells efficiently mediate humoral immunity. Therefore, we analyzed the titers of anti-SARS-CoV-2 IgG (IgG) at disease onset as well as the titers of WT and alpha neutralizing antibodies and correlated these results with the NAb titers of the vac-healthy cohort at Tb. In other words, we performed two neutralization assays, where we tested the neutralizing capacity of the vac-healthy control and VBI diseased cohorts against the whole virus (hereafter referred to as WT NAb and alpha NAb) and against the S protein only, hereafter referred to as S-WT NAb and S-alpha NAb, respectively. The purpose of performing both assays is to indirectly evaluate the individuals’ immunogenicity against the S protein by comparing the neutralizing capacity induced against the whole virus, including M and N proteins.

**Figure 3:**
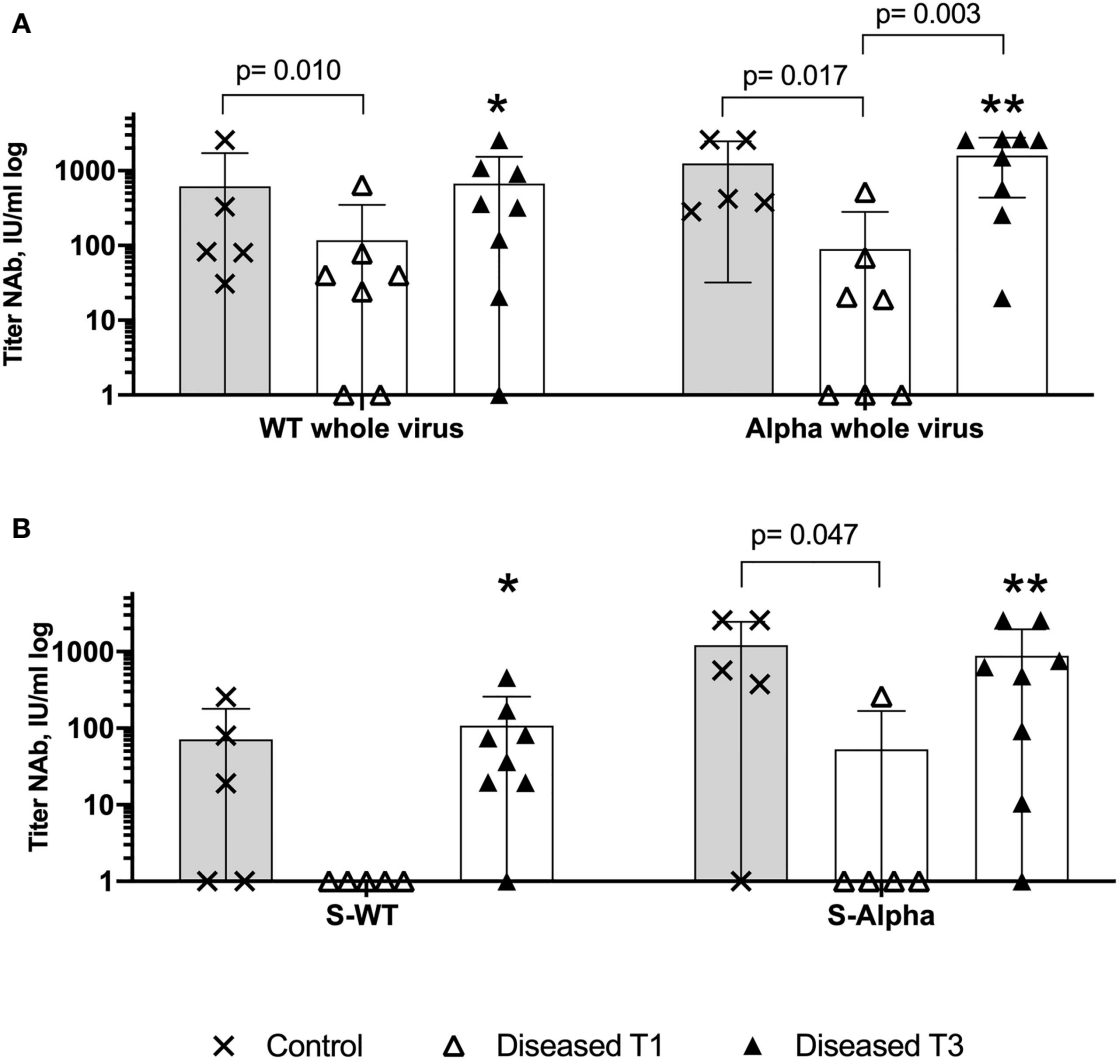
Insufficient WT and Alpha neutralizing antibodies production on disease onset. Anti-Sars-CoV-2 S protein and whole virus binding neutralizing antibodies assessed with WT virus hCoV-19 and VOC B.1.1.7 (Alpha). Comparison of the relative titers of SARS-CoV-2 neutralizing antibodies with the 50% neutralization dose among VBI, at T1 and T3, and the control cohort. **(A)** There is a significantly higher neutralizing potential of the control cohort against the Alpha whole virus, WT whole virus and **(B)** against the S-Alpha at disease onset, indicating failure of the anti-SARS-CoV-2 vaccination to generate protective humoral immunity among the diseased cohort. ⁎ (WT whole virus VBI T3 versus S-WT VBI T3), ⁎⁎ (Alpha whole virus VBI T3 versus S-Alpha T3). Increased neutralizing antibodies titers at T3, with Alpha and WT whole virus NAb titers are statistically significantly higher compared to S-WT and S-Alpha NAb at T3, implying reduced immunogenicity of the diseased cohort against the SARS-CoV-2 Spike (WT NAb versus S-WT NAb p=0.015, Alpha NAb versus S-Alpha NAb p=0.015). Paired data was compared with Wilcoxon matched pairs test, whereas unpaired data with Mann-Whitney-test. P<0.05 was considered significant, only significant p values are documented in the figures.

All individuals in the vac-healthy group showed efficient neutralizing capacity against the WT and more robust titers against the alpha variant (**Figures 3A** and **S5** Column A). The vac-healthy study group showed similar titers of alpha NAb and S-alpha NAb (**Figures 3A, B**).

Seventy-five percent (n=6) of the VBI group showed IgG titer, and 25% (n=2) showed no IgG titer (P1+P6) (**Figure S5D** and **Table S1**). VBI patient P5 (**Table S1** and **Figures S5** 1B & 2B) showed positive IgG titers but without neutralizing capacity. The neutralizing capacity, characterized by the absolute number of individuals with neutralizing antibodies, against S-WT NAb and S-alpha NAb among the VBI at disease onset was 0% and 12.5%, respectively. The vac-healthy cohort had neutralizing capacities of 100% and 80% against S-WT NAb and S-alpha Nab, respectively. Our analysis of the titers of the neutralizing antibodies showed higher S-WT neutralizing titers for the vac-healthy cohort than the VBI diseased cohort at T1 (disease onset), but the difference was not statistically significant. This is in contrast to the S-alpha neutralizing titers of the vac-healthy cohort, which were significantly higher than those of the VBI diseased cohort at disease onset (**Figures 3B** and **S5** Rows 2 and 4).

Next, we evaluated the neutralizing capacity at disease onset against the whole virus. The vac-healthy cohort presented significantly higher neutralizing titers against both WT and alpha than the VBI diseased cohort at T1 (**Figures 3A** and **S5** Rows 1 and 3). Among the VBI patients, neutralization capacity for S-WT NAb and S-alpha Nab could not be demonstrated, but positive WT NAb and alpha NAb were observed at T1 (**Figures 3** and **S5** column B). In the course of COVID-19, the VBI diseased subjects were able to mount neutralizing antibodies with values slightly higher than or comparable to the vac-healthy cohort for S as well as for the whole virus (**Figures 3** and **S5** Column C). We compared the T1 and T3 timepoints and detected significantly increased values of neutralizing antibodies against the whole alpha variant. WT NAb was also increased at T3, but without reaching statistical significance. In addition, the neutralizing potential against the whole virus was significantly higher at T3 for both WT and alpha variants compared to S-WT NAb and S-alpha NAb, respectively (**Figure 3** ⁎ (WT whole virus VBI T3 versus S-WT VBI T3), ⁎⁎ (Alpha whole virus VBI T3 versus S-Alpha T3).

### Lymphopenia in SARS-CoV-2 VBI: Similar Absolute Lymphocyte Counts Among the VBI and No-Vac COVID-19 Patients

Beside the cytokine storm syndrome, lymphopenia is a hallmark of SARS-CoV-2 infection. It has been correlated with severe and critical COVID-19 disease, and T cell migration to the sites of infection is considered as one of the significant factors of lymphocyte depletion from the circulation (22). To exclude the influence of lymphopenia in clinical follow up on our results, we compared the absolute lymphocyte counts among the VBI cohort between T1 and T3. The VBI patients show similar absolute lymphocyte counts on T1 and T3 (1.015/nl and 1.1/nl respectively, **Table S1**). As expected, the absolute lymphocyte counts of the critically ill patients were lower at T1 compared to the severely and moderately diseased patients without reaching statistical significance. However, the limited available values should be taken into consideration when interpreting the results. Next, we compared the absolute lymphocyte counts of VBI patients versus an unvaccinated wildtype SARS-CoV-2 diseased (no-vac COVID) cohort at T1. The median absolute lymphocyte count on T1 among the no-vac COVID patients was 0.98/nl (range 0.66-1.9), which was not significantly different compared to VBI patients (**Table S3**).

### No Vaccination Advantage in VBI Group Compared to No-Vac COVID Group With Severe and Critical Disease

**Figure 4:**
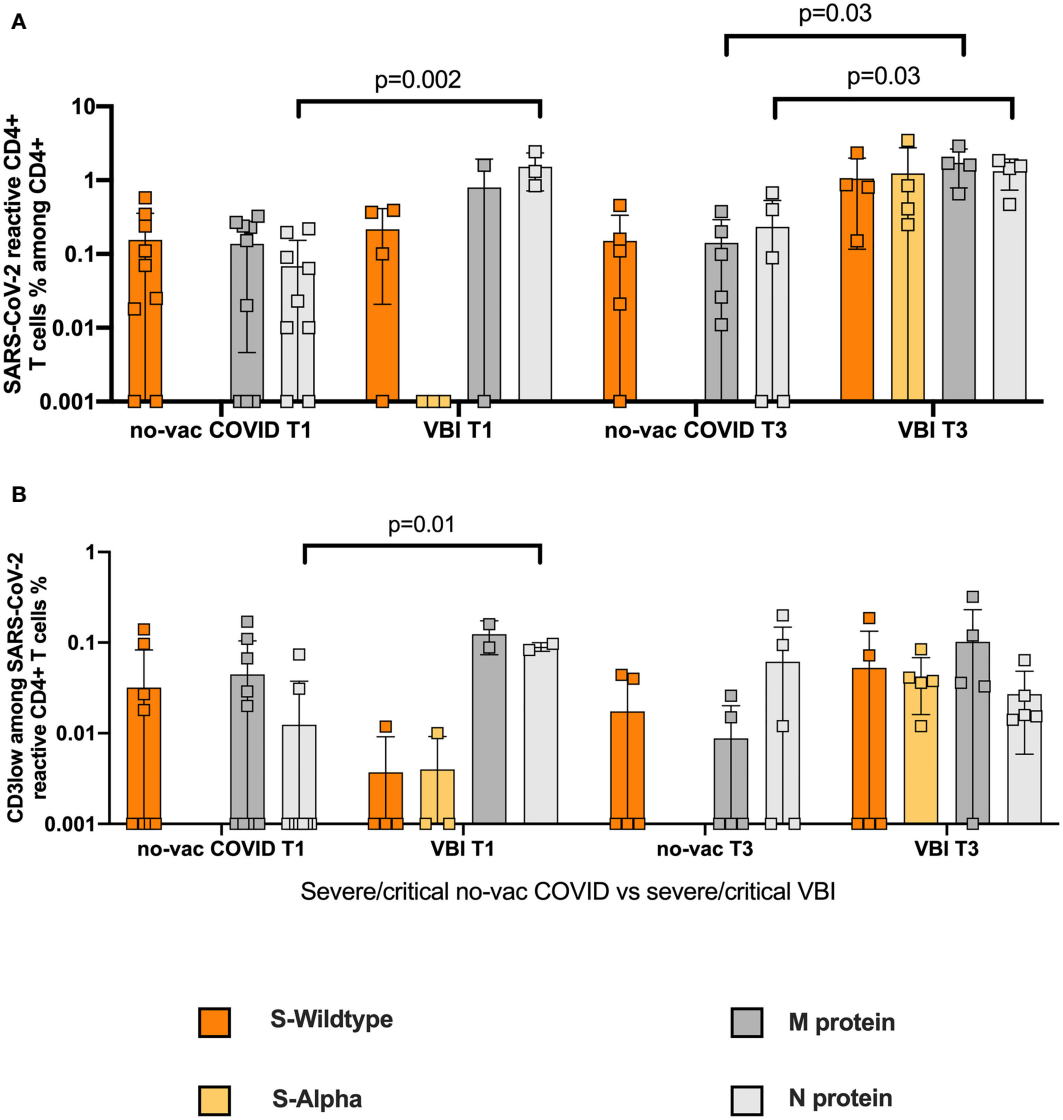
No vaccination advantage in VBI group compared to no-vac COVID group with severe and critical disease. Comparison of SARS-CoV-2 reactive CD4+ T cell response among the severe and critical VBI patients versus the severe and critical no-vac COVID-19 patients. **(A)** Frequencies of WT S- and M-reactive CD4+ T cells at T1 and T3 for the two cohorts are depicted **(B)** Comparison of CD3low populations of the unvaccinated COVID-19 cohort compared to the VBI patients.

To assess a possible advantage of the vaccination in severe and critical VBI, we compared the SARS-CoV-2 reactive CD4+ T cell response among the severe and critical VBI patients versus the severe and critical no-vac COVID-19 patients (**Table S3**). We demonstrate similar frequencies of WT S-reactive CD4+ T cells (**Figure 4A**) at T1 between the two cohorts. However, the analysis of the CD3low populations revealed WT S-reactive CD4+ T cells with higher avidity among the COVID-19 cohort compared to the VBI patients, suggesting there is no vaccination advantage among the VBI cohort (**Figure 4B**). At T3, both cohorts generate SARS-CoV-2 reactive CD4+ T cell response with high avidity against the tested proteins.

### Differentiated Humoral and Cellular Response in Moderate and Critical VBI

It is currently accepted that the magnitude of humoral and cellular immune response in SARS-CoV-2 infection defines COVID-19 disease severity (22), therefore we wondered if there are differences in immune response between moderate and critical VBI. We compared the frequencies of SARS-CoV-2 reactive CD4+ T cells and their avidity in critical and severe VBI patients compared to the moderate VBI patients (**Figure S6A**). The critical and severe VBI patients presented higher frequencies of SARS-CoV-2 reactive CD4+ T cells against all four proteins. For the high avidity (CD3lymplow) CD4+ reactive T cells against all four proteins no differences were found between the severe and critical ill VBI patients compared to the moderate VBI group. Analysis of the humoral component (**Figure S5**-3B) revealed higher titers of NAb against the whole alpha variant at T1 in the two moderate VBI patients compared to severe and critical VBI.

### Low Frequencies of Pre-Existing SARS-CoV-2 Reactive T Cells With Low Avidity Among No-Vac Unexposed Controls

**Figure 5:**
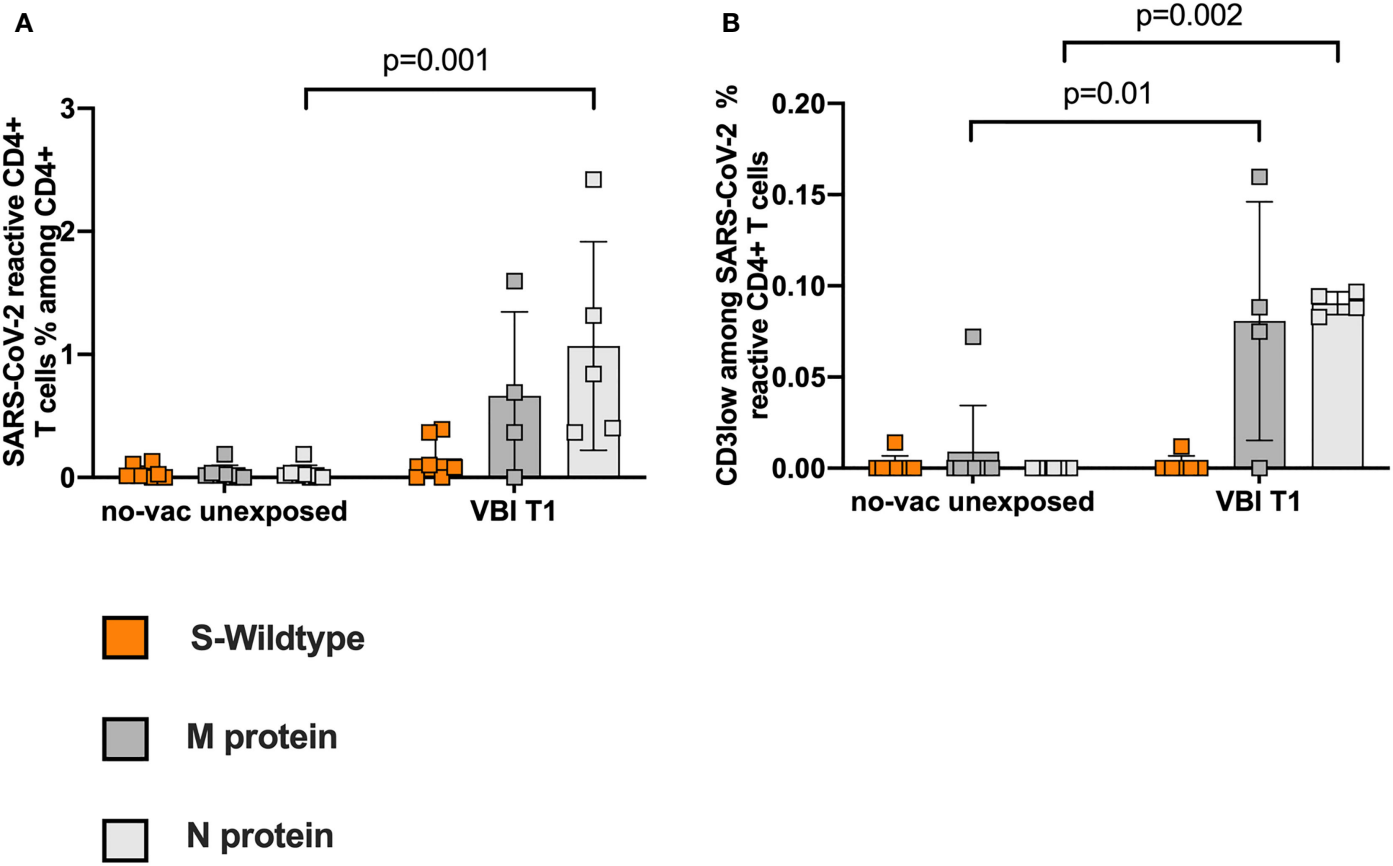
Low frequencies of pre-existing SARS-CoV-2 reactive T cells with low avidity in the unvaccinated unexposed controls. PBMCs from 8 individuals, unexposed to SARS-CoV-2, were analyzed for pre-existing SARS-CoV-2 reactive CD4+ T cell response. **(A)** Low frequencies of pre-existing cross-reactive CD4 reactive T cells compared to VBI patients at T1. **(B)** The avidity of M- and N-CD3low populations is significantly lower among the no-vac unexposed cohort.

Pre-existing SARS-CoV-2 immunity has been detected by independent working groups (32, 33), however the current data is contradictory regarding its function upon infection or vaccination (29, 35). To evaluate the influence of pre-existing SARS-CoV-2 reactive CD4+ T cells on our findings, we used PBMCs from 8 individuals not exposed to SARS-CoV-2 (**Table S3**). The unexposed and unvaccinated cohort is referred to hereafter as no-vac unexposed. We demonstrate low frequencies of pre-existing cross-reactive CD4 reactive T cells compared to VBI patients on T1 (**Figure 5A**). The avidity of M- and N-CD3low populations was significantly lower among the no-vac unexposed cohort (**Figure 5B**).

## Discussion

Herein, we present a profiling of cellular and humoral immunity in a small cohort of 8 patients who developed COVID-19 disease, despite being inoculated twice against the SARS-CoV-2 virus. Our data suggests a failure of SARS-CoV-2 mRNA inoculation to induce protective cellular and humoral immunity reactive against the S protein at disease onset upon infection with the alpha SARS-CoV-2 VOC. However, VBI diseased patients were able to generate a delayed S protein cellular and humoral immune response. Based on our findings, which demonstrated higher frequencies of N- and M-reactive T-cell responses at disease onset, we hypothesize that our VBI diseased cohort presents with decreased immunity against SARS-CoV-2 WT and alpha S protein after the prime-boost COVID-19 vaccination.

We detected a WT S-reactive CD4+ T-cell response with low avidity at disease onset, implying a failure of cross-reactivation upon infection with the alpha variant. The concept of immune evasion in the frame of SARS-CoV-2 infection is gaining increasing attention, as VOCs, including alpha, encompass mutations that can facilitate escape from the vaccine-induced adaptive immunity (32, 33). Accumulating data imply that mutations could be neutral or result in either loss or gain of predicted epitopes depending on HLA type (12, 34, 36, 37). Furthermore, preliminary results suggest that the N501Y mutation leads to poorer presentation across the majority of MHC-II alleles, implying that the N501Y mutation may not only diminish the binding of antibodies to the receptor-binding domain, but also interfere with their production by weakening the cooperation between CD4+ T cells and B cells (38). As a possible explanation for the poor alpha SARS-CoV-2-reactive CD8+ T-cell response at disease onset found in our study, independent research groups highlighted previously the capacity of SARS-CoV-2 to evade cellular adaptive immune responses through sporadically emerging mutations in MHC-I epitopes and therefore escape the CD8+ T-cell response (39, 40).

Among the VBI diseased cohort, we observed a failure to generate high-avidity WT and alpha S-reactive T cells accompanied by the absence of neutralizing antibodies against the spike protein at disease onset. In contrast, a robust SARS-CoV-2 CD4+ T-cell response against the N and M proteins at disease onset was observed. Findings from others and our group prove that T-cell responses are focused not only on S but also on M, N, and other open reading frames (32–35, 41). Our results indirectly indicate reduced immunity against the spike protein in VBI patients. An important challenge arises as the mutation of virus proteins might alter the antigenicity of the virus and possibly affect human immune responses to the epitopes. The limited neutralizing effect in our cohort by a vaccine using the S protein as the only antigen suggests that epitopes of M and N proteins might also be considered for the design of the next generation of SARS-CoV-2 vaccines (34, 42).

A limitation of our study is the small number of patients, and the cohorts also demonstrated a significant age difference between the vaccinated healthy control cohort and the VBI cohort. Overall, our study analyzed the SARS-CoV-2 T-cell response and humoral immunity in severe and critical VBI. We demonstrate the absence of a SARS-CoV-2-reactive CD4+ T-cell response against the alpha variant in VBI patients, an ineffective humoral compartment and low avidity of WT SARS-CoV-2-reactive CD4+ T cells at disease onset as the leading immune pathological factors of VBI in our patient cohort. Furthermore, our findings are derived from measurements of circulating blood and the lack or diminished number of certain immune cell populations could reflect their migration to lymph nodes, lungs and other compartments.

Our observations do not undermine the importance of vaccination strategies. They support continued surveillance of VBI cases to help target VOCs for inclusion in new or booster SARS-CoV-2 vaccines (20, 43). Furthermore, our findings support the effort to develop a new generation of SARS-COV-2 vaccines with additional antigens and T cell epitopes—even a pancoronavirus vaccine—to prevent new pandemic or endemic outbreaks due to new variants or emerging coronaviruses (44, 45).

## Data Availability Statement

The original contributions presented in the study are included in the article/**Supplementary Material**. Further inquiries can be directed to the corresponding author.

## Author Contributions

KP and NB participated in research design. KP, MK, BH, HR, LF, CQ, and FS participated data curation and sample acquisition. KP, NB, TR, US, MA, CT, and SD participated in the writing of the paper. NB, OW, TW, and US participated in funding acquisition and project administration. KP, TM, SP, CM, SS, MA, and OA participated in the performance of the research. NB, TW, OW, and US contributed new reagents or analytic tools. KP, MA, US, and CT participated in data analysis. All authors contributed to the article and approved the submitted version.

## Funding

This work was supported by grants of Mercator Foundation, EFRE grant for COVID.DataNet.NRW,AiF grant for EpiCov, and BMBF for NoChro (FKZ 13GW0338B).

## Conflict of Interest

The authors declare that the research was conducted in the absence of any commercial or financial relationships that could be construed as a potential conflict of interest.

## Publisher’s Note

All claims expressed in this article are solely those of the authors and do not necessarily represent those of their affiliated organizations, or those of the publisher, the editors and the reviewers. Any product that may be evaluated in this article, or claim that may be made by its manufacturer, is not guaranteed or endorsed by the publisher.

## Abbreviations

VOC, variant of concern; VBI, vaccine breakthrough infection; Vac-healthy, vaccinated healthy cohort; S, spike; WT, wild type; PBMC, peripheral blood mononuclear cell; Ct, cycle threshold; RT–PCR, real-time polymerase chain reaction; IL2, interleukin 2; TNFα, tumor necrosis factor α; IFNγ, interferon γ; NAb, neutralizing antibody; No-vac COVID, unvaccinated wildtype SARS-CoV-2 diseased cohort; No-vac unexposed, unvaccinated unexposed to SARS-CoV-2 cohort.
