# Supplementary material for: Immune Response in Moderate to Critical Breakthrough COVID-19 Infection After mRNA Vaccination
Source: Front Immunol. 2022 Jan 25;13:816220. doi: 10.3389/fimmu.2022.816220 (PMC8821964; doi:10.3389/fimmu.2022.816220)
Supplement: Supplementary file 1 [file DataSheet_1.docx]

**Supplemental Table of Contents**

**Supplementary Tables**

**Table S1**. Demographic and virological characteristics of the VBI cohort

**Table S2.** Clinical characteristics of the VBI group

**Table S3.** Synopsis of the demographic characteristics of study participants

**Table S4:** Fluorochrome coupled antibodies and fluorescent dye for analysis of SARS-CoV-2 reactive T cells

**Legends of Supplementary Figures**

**Figure S1.** Flow cytometry gating strategy for identification and quantification of SARS-CoV-2 reactive T cells.

**Figure S2.** Robust SARS-CoV-2 reactive CD4+ and CD8+ T cell response against S-WT and S-Alpha among the vac-healthy cohort.

**Figure S3.** Failure to mount monofunctional Alpha S-reactive CD4+ T cells at VBI disease onset.

**Figure S4.** **Re-evaluation of the reactive CD4+ T cell response excluding P2, P4 and P6 VBI patients**

**Figure S5.** Descriptive presentation of neutralizing antibodies against whole virus WT, S-WT, whole virus Alpha and S-Alpha individualized per participant and disease severity.

**Figure S6. Reactive CD4+ T cell response differs depending on disease severity**

**Table S1**. Demographic and virological characteristics of the VBI cohort

|  | **Age (years)** | **Gender** | **Disease Severity (WHO classification)** | **Vaccine type** | **Days from 2^nd^ vaccination to**  **first positive RT-PCR Test** | **Ct value of 1^st^ positive**  **RT-PCR Test** | **Sequencing** | **Anti-SARS-CoV-2 IgG on COVID-19 diagnosis (AU/ml)** | **Comorbidities** | **Lymphocyte absolute counts/nl (normal range 1-4/nl)** | |
| --- | --- | --- | --- | --- | --- | --- | --- | --- | --- | --- | --- |
|  |  |  |  |  |  |  |  |  |  | **T1** | **T3** |
| **P1** | 78 | Female | Severe | mRNA-1273 | 60 | 14 | S-N501Y positive  H69/V70 positive  S-E484K negative | negative | Obesity, arterial hypertension | 1.50 | - |
| **P2** | 81 | Male | Severe | BNT162b2 | 10 | 14,73 | S-N501Y positive  H69/V70 positive  S-E484K negative | 130 | COPD, coronary heart disease, history of cancer | 1.14 | 1.90 |
| **P3** | 28 | Female | moderate | BNT162b2 | 50 | 19,77 | S-N501Y positive  H69/V70 positive  S-E484K negative | 434 | Nicotine abuse | 2.11 | - |
| **P4** | 81 | Male | Severe | mRNA-1273 | 12 | 15,62 | S-N501Y positive  H69/V70 positive  S-E484K negative | 100 | Terminal kidney failure | - | 1.10 |
| **P5** | 82 | Female | moderate | BNT162b2 | 75 | 20,72 | S-N501Y positive  H69/V70 positive  S-E484K negative | 227 | Terminal kidney failure, coronary heart disease, diabetes | - | - |
| **P6** | 80 | Male | critical | BNT162b2 | 11 | 20,18 | S-N501Y positive  H69/V70 positive  S-E484K negative | negative | Terminal kidney failure, arterial hypertension | 0.40 | - |
| **P7** | 81 | Female | critical | BNT162b2 | 20 | 25 | S-N501Y positive  H69/V70 positive  S-E484K negative | 157,4 | Diabetes | 0.89 | - |
| **P8** | 80 | Female | critical | BNT162b2 | 43 | 21 | S-N501Y positive  H69/V70 positive  S-E484K negative | 300 | History of Cancer | 0.84 | 1.10 |
| **Median** | 80.5 | N/A | N/A | N/A | 31,5 | 19,97 | N/A | 143,7 | N/A | 1.015 | 1.10 |
| **Min** | 28 | N/A | N/A | N/A | 10 | 14 | N/A | 0 | N/A | 0.40 | 1.10 |
| **Max** | 82 | N/A | N/A | N/A | 75 | 25 | N/A | 434 | N/A | 2.11 | 1.90 |

**Table S2.** Clinical characteristics of the VBI group

|  | **Complications** | **Outcome** | **Drug therapy** | **Oxygen therapy/ Invasive Ventilation** | **Duration of**  **hospitalization (days)** |
| --- | --- | --- | --- | --- | --- |
|  |  |  |  |  |  |
| **P1** | Septic Encephalopathy | Discharge | Remdesivir/ Antibiotics  Dexamethasone | Oxygen therapy | 18 |
| **P2** | None | Discharge | Remdesivir/ Antibiotics  Dexamethasone | Oxygen therapy | 18 |
| **P3** | None | Recovery | None | none | 0 |
| **P4** | None | Discharge | Carisivimav/ Imdevimab  Antibiotics | None | 27 |
| **P5** | None | Recovery | None | None | 0 |
| **P6** | Multiorgan failure | Death | Antibiotics  Dexamethasone | Invasive | 9 |
| **P7** | Multiorgan failure | Death | Remdesivir/ Antibiotics  Dexamethasone | Invasive | 39 |
| **P8** | none | Discharge | Remdesivir/ Antibiotics  Dexamethasone | Invasive | 45 |
| **Median** | N/A | N/A | N/A | N/A | 18 |
| **Min** | N/A | N/A | N/A | N/A | 0 |
| **Max** | N/A | N/A | N/A | N/A | 45 |

| **Cohorts** | **Abbreviation** | **Number (N)** | **Median age- years (range)** | **Male % (N)** | **Sample**  **Timepoints** | **Disease Severity -WHO classification % (N)** | **Lymphocyte absolute counts on T1 (normal range 1-4/nl) median (range)** |
| --- | --- | --- | --- | --- | --- | --- | --- |
| **Vaccine breakthrough infection (B.1.1.7 infection)** | VBI | 8 | 80.5 (28-82) | 38% (3) | T1, T2, T3* | Moderate 24% (2)  Severe 38% (3)  Critical 38% (3) | 1.01 (0.4-2.11) |
| **Healthy - 2x BNT162b2 vaccinated cohort** | Vac-healthy | 5 | 31 (27-34) | 20% (1) | Ta, Tb** | n/a | unknown |
| **Unvaccinated COVID-19 cohort**  **(Wildtype infection)** | No-vac COVID | 14 | 72 (44-89) | 64% (9) | T1, T3 | Moderate 36% (5)  Severe 36% (5)  Critical 28% (4) | 0.98 (0.66-1.90) |
| **Unexposed unvaccinated cohort (recruitment 2015-2017)** | No-vac unexposed | 8 | 52.5 (42-63) | 50% (4) | Ta | n/a | unknown |

**Table S3.** Synopsis of the demographic characteristics of study participants

* T1: third day since SARS-CoV-2 diagnosis, T2: 7 days since the diagnosis, T3: 14 days since T1.

**Ta: before 1^st^ vaccination or pre-pandemic, Tb: 14 days after the 2^nd^ vaccination.

**Table S4:** Fluorochrome coupled antibodies and fluorescent dye for analysis of SARS-CoV-2 reactive T cells

| **Antibodies or fluorescent dye** | **Fluorochrome** | **Source** | **Cat. Nr.** |
| --- | --- | --- | --- |
| Fixable Viability-Dye | eFluor780 | eBioscience | 65-0865-14 |
| anti CCR7 (clone G043H7) | PerCP-Cy5.5 | BioLegend | 353220 |
| anti CD4 (clone OKT4) | A700 | BioLegend | 317426 |
| anti CD8 (clone RPA-T8) | V500 | BD Biosciences | 560775 |
| anti CD45RA (clone HI100) | BV605 | BioLegend | 304134 |
| anti Granzyme B (clone GB11) | FITC | BioLegend | 515403 |
| anti IL2 (clone MQ1-17H12) | PE | BioLegend | 500307 |
| anti CD185(CXCR5) (clone MP4-25D2) | PE-Dazzle594 | BioLegend | 356927 |
| anti CD137 (4-1BB) (clone 4B4-1) | PE-Cy7 | BioLegend | 309818 |
| anti CD154 (CD40L) (clone 24-31) | A647 | BioLegend | 310818 |
| anti TNFα (clone MAb11) | eFluor450 | eBioscience | 48-7349-42 |
| anti IFNγ (clone 4S.B3) | BV650 | BioLegend | 502538 |
| anti CD3 (clone OKT3) | BV785 | BioLegend | 317330 |

**Supplementary Figures & Legends**


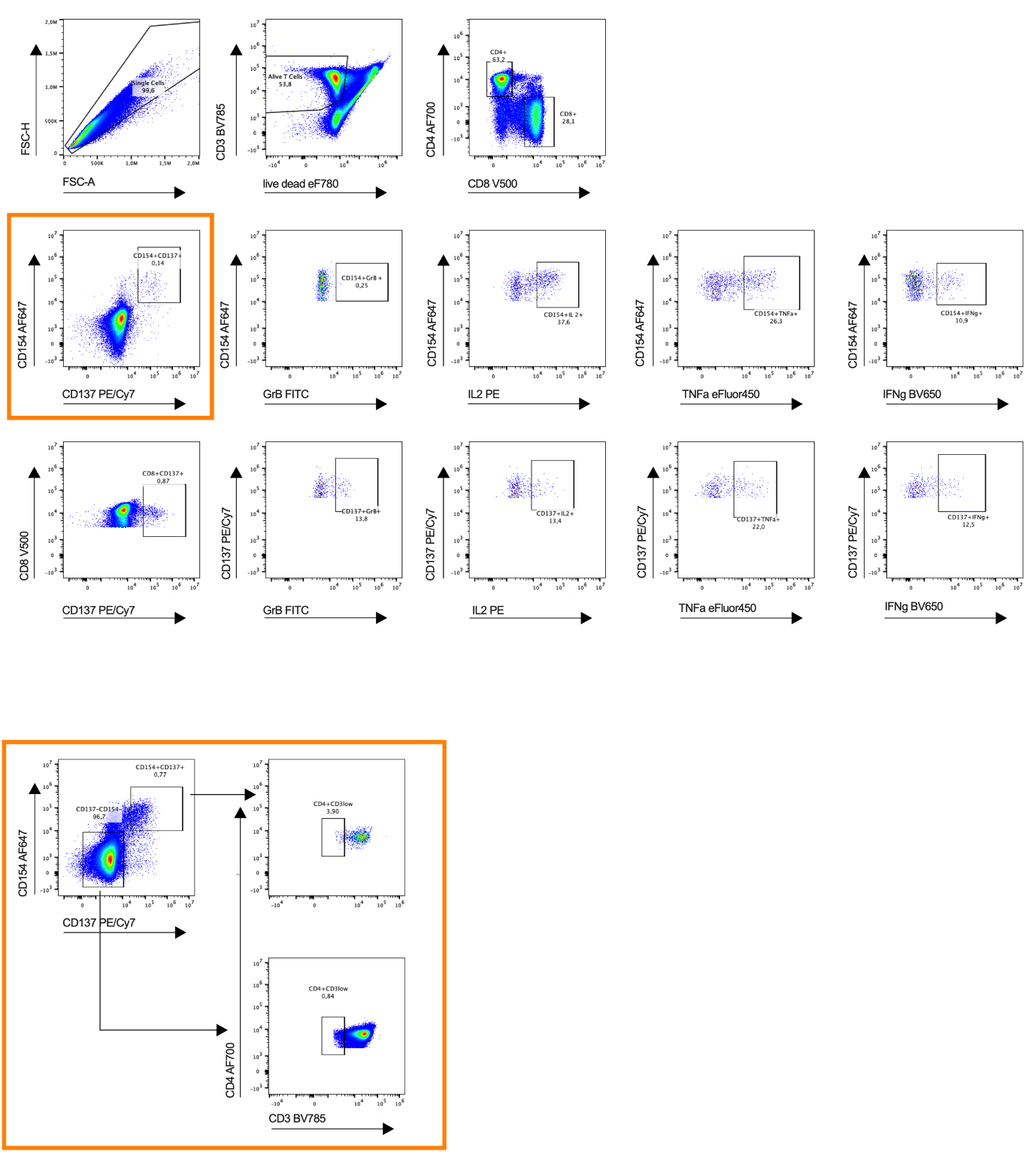


**Figure S1. Flow cytometry gating strategy for identification and quantification of SARS-CoV-2 reactive T cells.** PBMCs were stimulated for 16 h with one of the following SARS-CoV-2 peptides spanning *in silico* predicted immunodominant parts of the; WT Spike SARS-CoV-2, B.1.1.7 D614G Spike mutant, the complete sequence of the N- or M-protein or left untreated as a control. After 2 h, Brefeldin A was added to the culture to block secretion of cytokines and effector molecules. Living single lymphocytes were analyzed for expression of CD3, CD4, and CD8. CD4+ T cells (orange boxes) were analyzed for the expression of CD154 and CD137. CD8+ T cells were analyzed for expression of CD137. Both CD4+ and CD8+ T cells were further analyzed for the production of cytokines IFNγ, TNFα, and IL2. Furthermore, (orange boxes) CD4+CD154+CD137+ and CD4+CD154-CD137- cells were analyzed for the expression of CD3low. Representative example of 8 patients with SARS-CoV-2 breakthrough infection, 5 healthy vaccinated individuals, 14 unvaccinated COVID-19 disease patients and 8 unexposed to SARS-CoV-2 unvaccinated individuals. Plots of a VBI study subject are depicted.


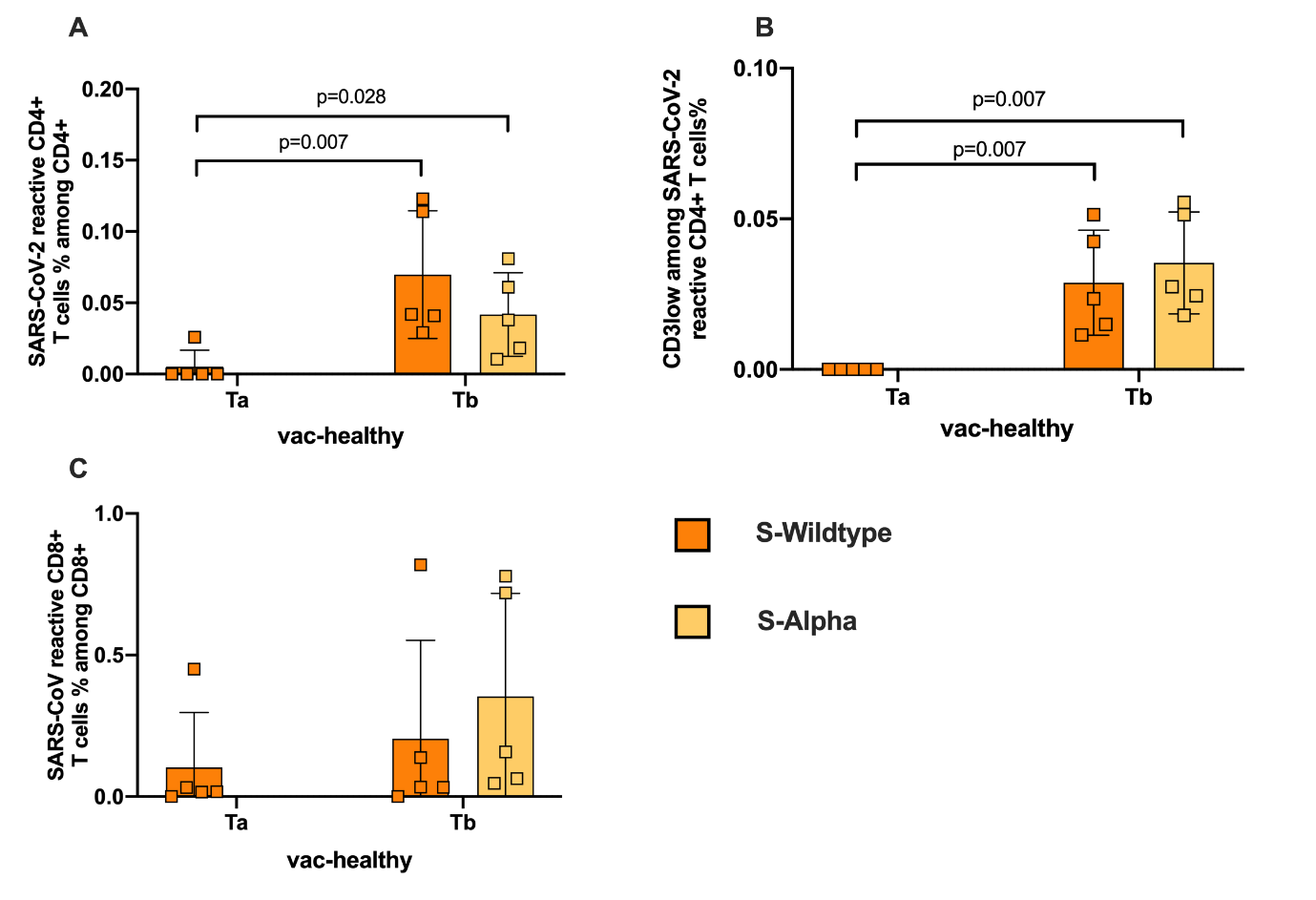


**Figure S2. Robust SARS-CoV-2 reactive CD4+ and CD8+ T cell response against S-WT and S-Alpha among the vaccinated healthy group.** Characterization of SARS-CoV-2 S-reactive CD4+ T cells in healthy subjects. Blood samples of 5 healthy vaccinated patients were stimulated with SARS-CoV-2 S-WT and S-Alpha peptides and analyzed by flow cytometry. A) A significant increase of S-protein-reactive CD4+ T cell response against the WT S-protein and the Alpha variant from Ta to Tb was detected. B) Statistically significant increase of CD3low response among CD4+CD154+CD137+ T cells against the two tested proteins in Tb was observed. C) The control cohort demonstrated stronger SARS-CoV-2 reactive CD8+ T cell frequencies against the S-WT and the Alpha variant at Tb compared to Ta, but without a significant difference.


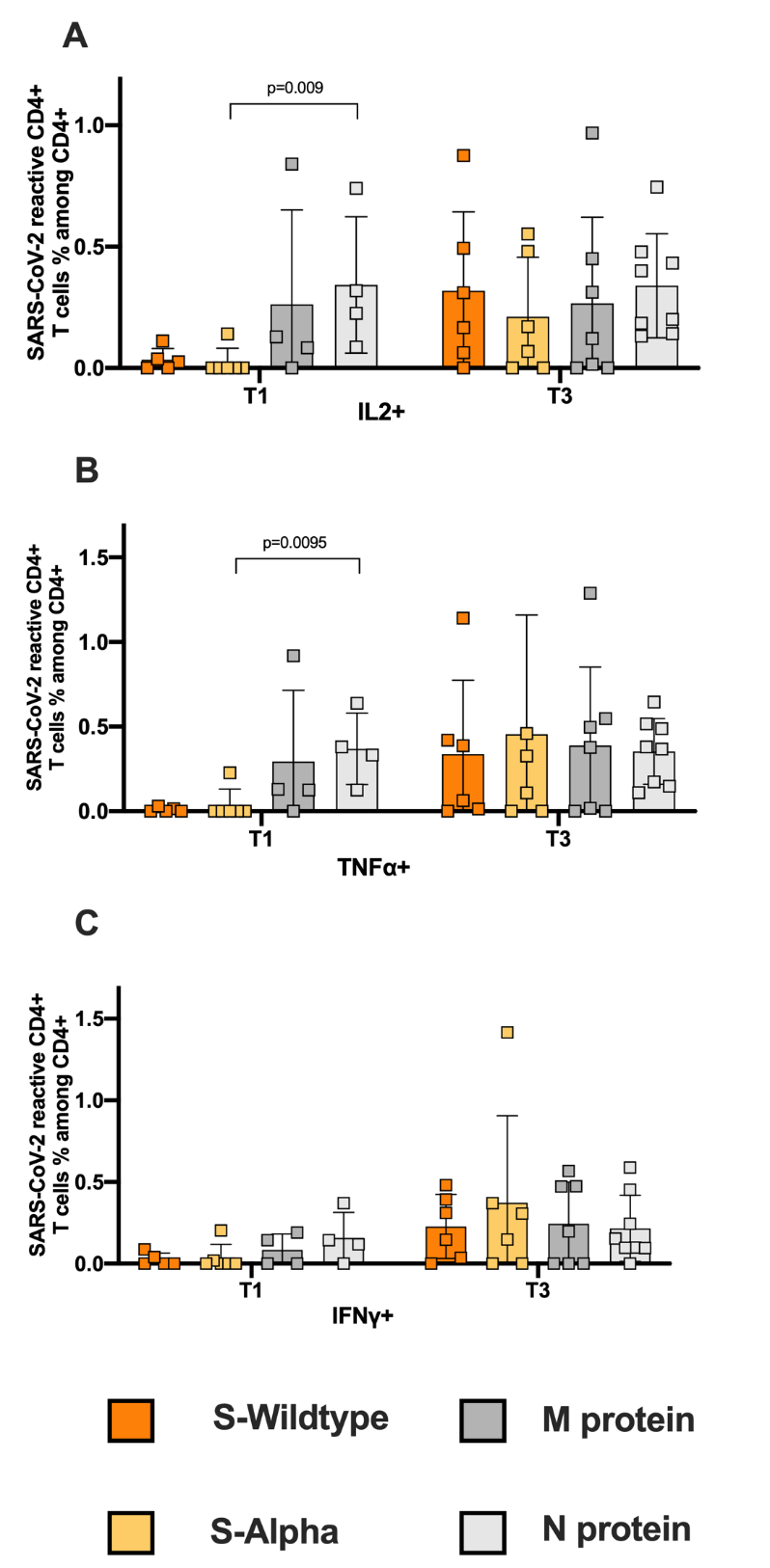


**Figure S3. Failure to mount monofunctional Alpha S-CD4+ T cells at VBI disease onset.** Characterization of the mono SARS-CoV-2 reactive CD4+ T cells among the VBI cohort, regarding the production of IL2 (A), TNFα (B), and IFNγ (C). Statistically significant higher frequencies for N-reactive CD4+IL2+ and N-reactive CD4+TNFα+ at VBI disease onset compared to S-Alpha reactive CD4+IL2+ and TNFα+ T cells. At T3 the VBI patients mount monofunctional reactive CD4+ T cells against all five tested proteins.


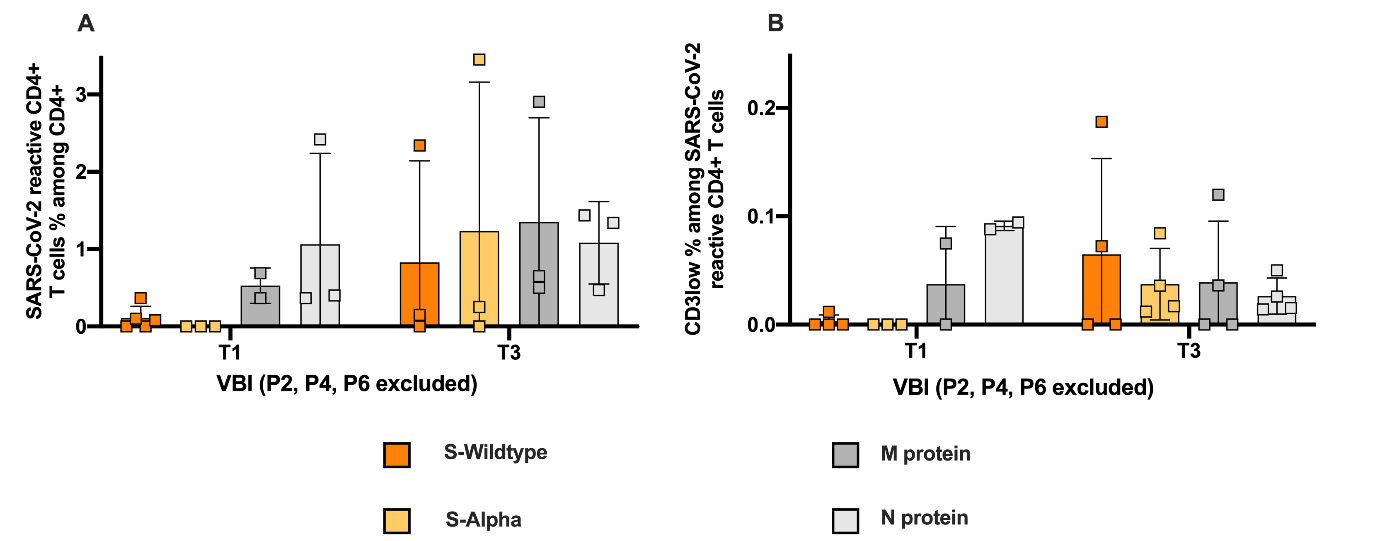


**Figure S4.** **Re-evaluation of the reactive CD4+ T cell response excluding P2, P4 and P6 VBI patients.** The data was reanalyzed excluding the 3 VBI patients, who were diagnosed with SARS-CoV-2 VBI infection 10 days after the 2nd vaccination. A and B: low frequencies of WT and Alpha S-reactive CD4+ T cells on T1 with low avidity, accompanied by higher frequencies of M- and N-reactive CD4+ T cells with high avidity at T1 were detected. At T3, reactive CD4+ T cell response against all proteins is detected and characterized by functional avidity.

**
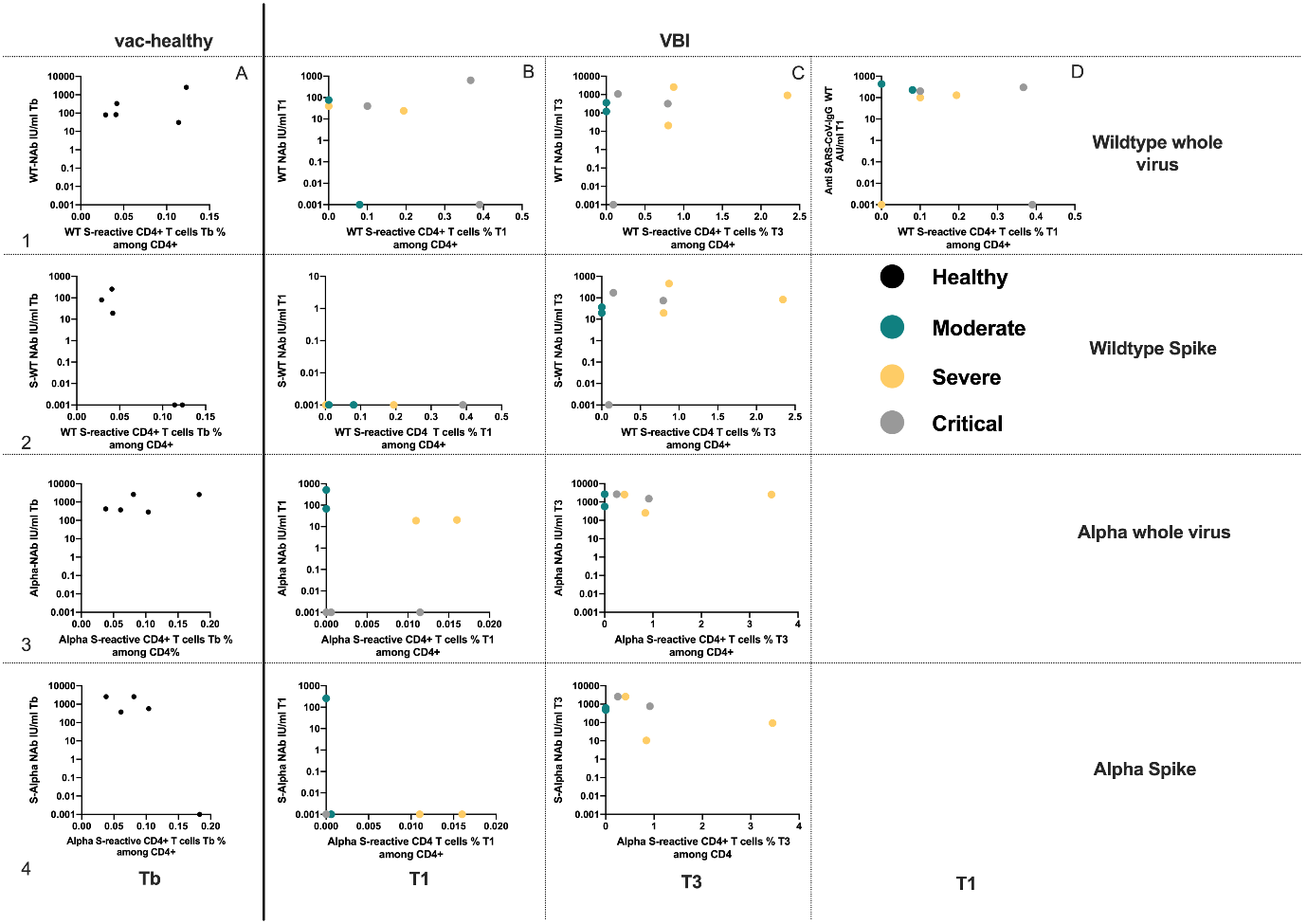
**

**Figure S5. Descriptive presentation of neutralizing antibodies against whole virus WT, S-WT, whole virus Alpha and S-Alpha individualized per participant and disease severity.** The horizontal lines categorize the neutralizing peptide or virus, whereas the colors the disease severity. Column A presents the control group, while columns B and C the diseased cohort at T1 and T3. (D1) Comparison of the relative titers of SARS-CoV-2 WT-specific IgG antibodies, measured by ELISA, to WT S-reactive CD4+ T cells at T1.


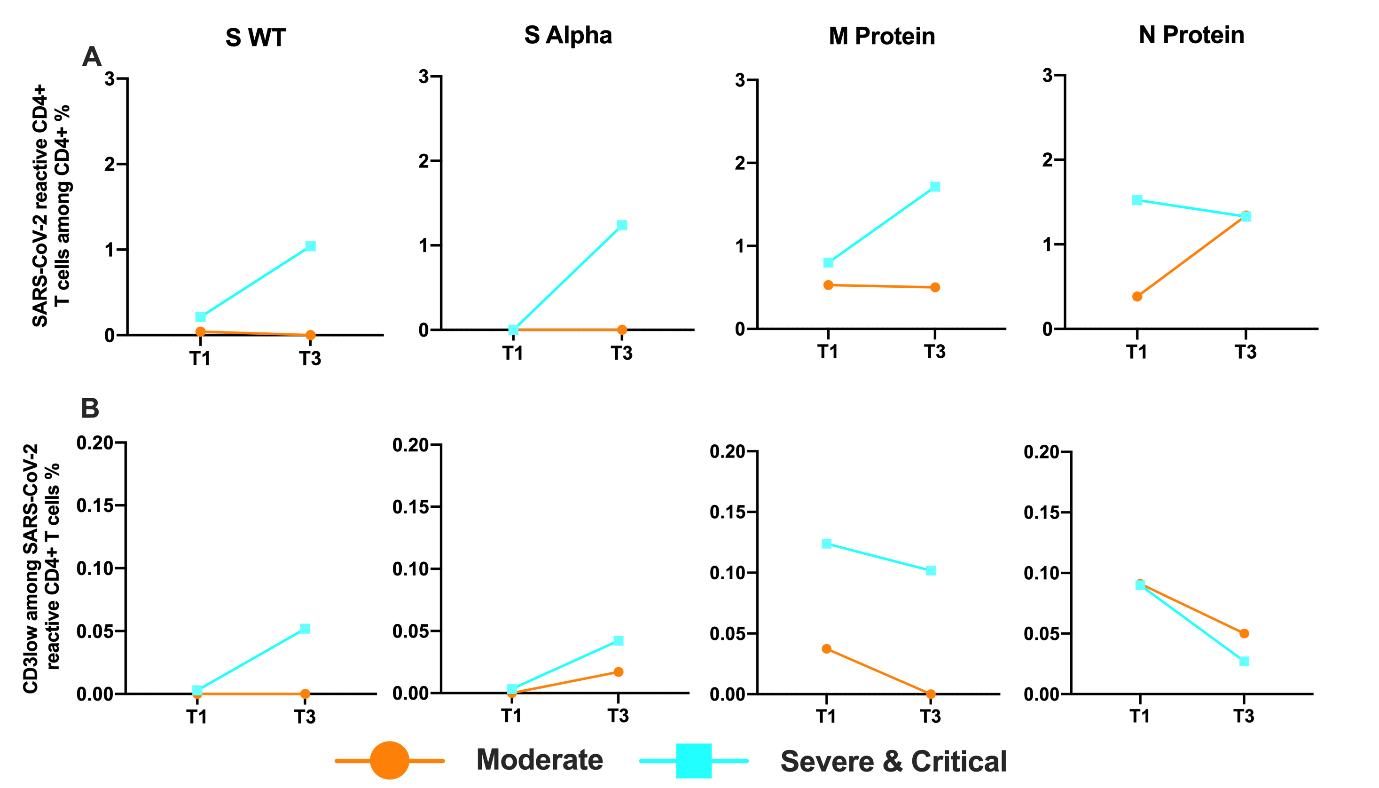


**Figure S6. Reactive CD4+ T cell response differs depending on disease severity.** The frequencies of SARS-CoV-2 reactive CD4 T cells and their avidity among critical and severe VBI patients versus the moderate VBI patients were compared. A) The critical and severe VBI patients show higher frequencies of SARS-CoV-2 reactive CD4+ T cells against all four proteins. B) The avidity of reactive CD4+ T cells is similar among the VBI patients, independent of the disease severity.
